# Emergency Admission Plasma D‐Dimer and Prothrombin Activity: Novel Predictors for Clinical Outcomes After Thrombectomy in Acute Ischemic Stroke With Large Artery Occlusion

**DOI:** 10.1111/cns.70267

**Published:** 2025-02-13

**Authors:** Shandong Jiang, Peizheng Guo, Linxin Cai, Cong Qian, Jun Yu, Liang Xu, Xu Li, Xianyi Chen, Fang Bing, Yuan Yuan, Zhongju Tan, Jing Xu, Jianru Li

**Affiliations:** ^1^ Department of Neurological Surgery The Second Affiliated Hospital of Zhejiang University School of Medicine Hangzhou China; ^2^ Department of Geriatrics The First Affiliated Hospital of Zhejiang University School of Medicine Hangzhou China

**Keywords:** acute ischemic stroke, D‐dimer, prothrombin activity, thrombectomy

## Abstract

**Background:**

The coagulation system is intrinsically linked to pathological mechanisms and progression of ischemic stroke. However, the role of preoperative coagulation function in determining the functional outcomes of acute ischemic stroke patients following large artery occlusion (AIS‐LVO) has not been extensively evaluated in peer‐reviewed literature.

**Methods:**

We utilized logistic regression analyses, complemented by the construction of receiver operating characteristic (ROC) curves, to identify significant predictive factors for poor prognosis following endovascular thrombectomy (EVT). Additionally, subgroup analyses were conducted to further assess the prognostic efficacy of coagulation function across different subgroups.

**Results:**

A total of 607 patients were enrolled, with 335 (55.19%) experiencing an unfavorable outcome. Multivariate regression analysis identified preoperative D‐dimer and PTA as independent predictors of 3‐month prognosis. After adjusting for confounders, elevated preoperative D‐dimer levels (≥ 715 mg/L), identified by cut‐off value, were a significant predictor of poor prognosis, with 2.51‐fold higher risk compared to the normal range. Conversely, elevated PTA levels (≥ 85.5%) were significantly and inversely associated with poor prognosis, indicating a reduced risk of 0.39 times. Furthermore, the combination of elevated D‐dimer and reduced PTA demonstrated a synergistic effect, markedly increasing the risk of poor outcomes in AIS‐LVO patients. Subgroup analyses revealed that failed recanalization, comorbid diabetes, and non–middle cerebral artery (MCA) occlusion significantly influence the predictive value of D‐dimer and PTA for clinical outcomes.

**Conclusion:**

Elevated admission D‐dimer and reduced PTA levels are independent predictors of poor prognosis in patients with AIS‐LVO, and there is a synergistic interaction between the two variables.

## Graphical abstract

Preoperative high D‐dimer but low PTA level were the independent predictors of 3 months' unfavorable prognosis for AIS‐LVO after calibration of confounders. Elevated admission D‐dimer (≥ 715 mg/L) and reduced PTA levels (< 85.5%) are independent predictors of poor prognosis for patients with AIS‐LVO, and there is a synergistic interaction between the two variables with higher odds ratio (aOR = 4.55, 95% CI 2.63–7.69).

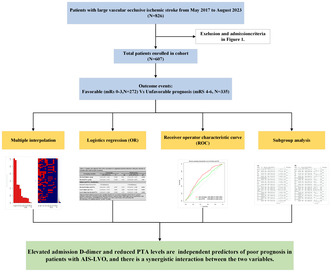

## 1 Introduction

Acute ischemic stroke (AIS) is the second most lethal and disabling disease worldwide, accounting for approximately 80% of all strokes. It severely impairs patients' survival and quality of life, leading to significant economic and social consequences [1]. Clinically, about one‐third of AIS patients are with acute large vessel occlusions (AIS‐LVO), which present with more severe clinical symptoms and higher rates of disability and mortality compared to non–large vessel occlusion strokes [2]. In 2015, five landmark randomized controlled trials (RCTs) established the superiority of endovascular thrombectomy (EVT) over intravenous thrombolysis (IVT) for the treatment of anterior circulation AIS‐LVO [3]. EVT, including endovascular contact aspiration and stent retriever techniques, has become a cornerstone therapeutic approach for AIS‐LVO, extending from anterior to posterior circulation cerebral infarctions [4]. Technological and material advancements have led to a marked increase in the recanalization rate among AIS‐LVO patients, rising from 58% to 65% in 2015 [3] to an impressive 83.1%–92% [5]. However, despite these improvements, the overall prognosis remains suboptimal, with a significant proportion of patients (47.6%–59.8%) failing to achieve a favorable outcome or functional independence post‐EVT [6, 7]. Therefore, it is important to explore the risk factors of unfavorable prognosis in patients with AIS‐LVO after EVT, which may help guide treatment decisions and the expectations of patients and their families.

In clinical practice, certain clinical features have been identified as predictors of poor prognosis in AIS patients post‐EVT, such as age [8], National Institutes of Health Stroke Scale (NIHSS) scores [9], hyperglycemia [10], dyslipidemia [11], and pass attempt exceeding three times during the procedure [12]. However, some of these indicators, like NIHSS or modified Rankin Scale (mRS) scores, are subject to variability due to differences in assessment across hospitals and individuals. Due to the clinical ubiquity and routine of preoperative testing, researchers have increasingly focused on the diagnostic and prognostic value of various auxiliary indices. These objective measures offer the potential for more standardized and reliable prognostic assessment, which is crucial for guiding treatment decisions and setting realistic expectations for patients and their families, ultimately aiming to improve patient outcomes following EVT [13, 14].

From a pathophysiological perspective, AIS is characterized by dynamic changes in the coagulation system during its acute phase. The modulation of thrombin generation and the coagulation cascade has been a longstanding target for both the treatment and prevention of AIS [15]. Elevated brain thrombin levels, detected in the infarct area following ischemia, are attributed to the breakdown of the blood–brain barrier (BBB) and the entry and synthesis of prothrombin in the brain [16], playing a significant role in the brain's pathological response to ischemic stroke. As part of the routine preoperative examination, coagulation function indicators, including D‐dimer, international normalized ratio (INR), prothrombin time (PT), activated partial thromboplastin time (APTT), prothrombin activity (PTA), thrombin time (TT), and fibrinogen (FIB), provide insights into both the extrinsic and intrinsic coagulation pathways [17], which may be closely associated with prognosis of AIS. Recent studies have begun to explore the relationship between these biomarkers and symptomatic intracerebral hemorrhage (sICH) after EVT [18], as well as their potential for more accurate AIS diagnosis [19]. However, these studies have primarily focused on isolated aspects of the coagulation and fibrinolysis process, neglecting a holistic view. To our knowledge, there is a gap in the literature regarding the exploration of the predictive effects of a range of coagulation function indicators on the prognosis of AIS patients post‐EVT. Therefore, the objective of our study was to investigate the association between coagulation function (D‐dimer, PT, INR, APTT, PTA, TT, and FIB) and modern and long‐term prognosis of patients with AIS‐LVO after EVT.

## 2 Methods

### 2.1 Study Design and Populations

**FIGURE 1:**
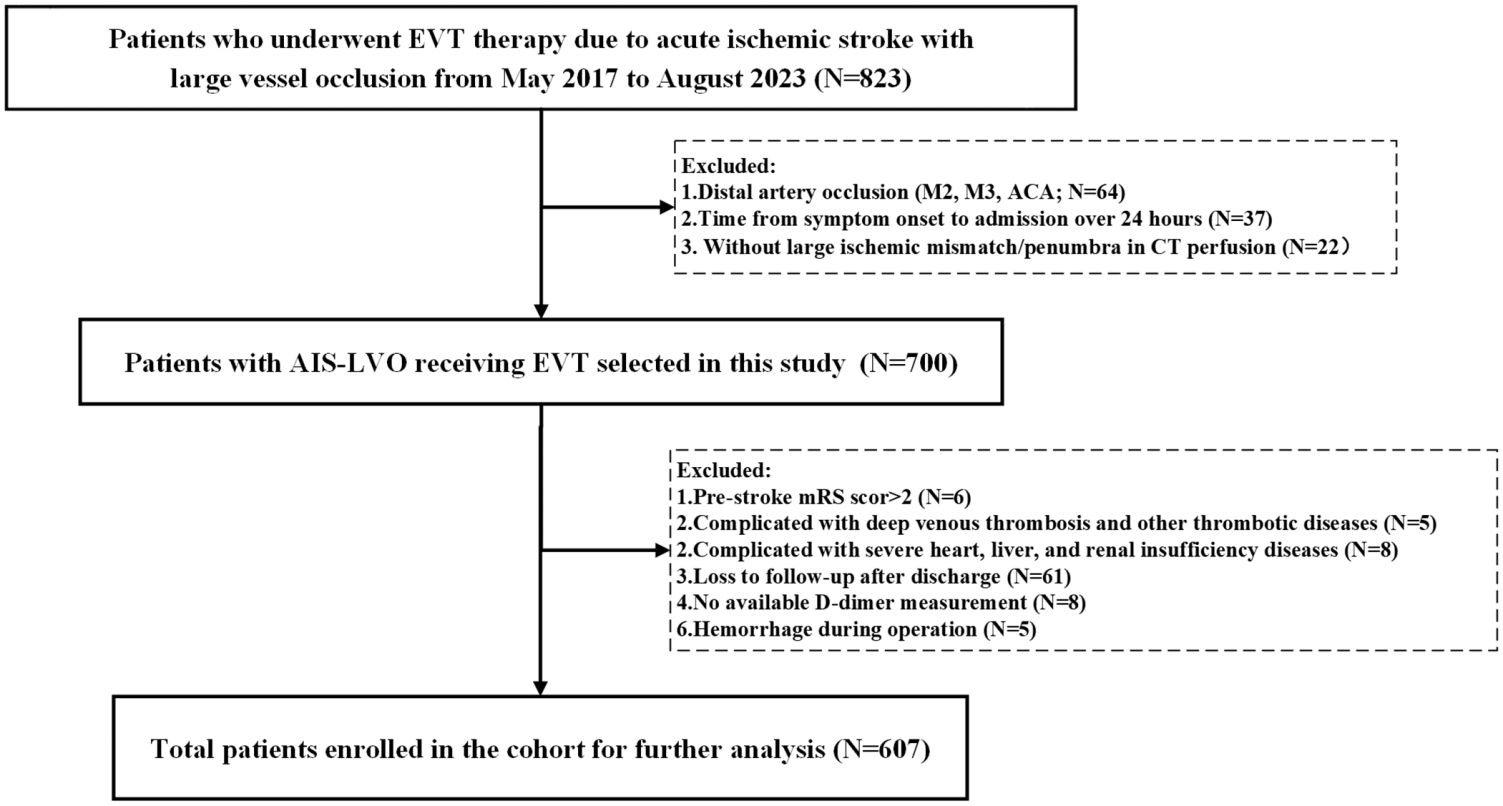
Flow chart of enrolled patients. ACA, anterior communicating artery; EVT, endovascular thrombectomy; ICA, internal carotid artery; IVT, intravenous thrombolysis; M2/M3, second/third segment of the middle cerebral artery; mRS, modified Rankin scale; PE, pulmonary thromboembolism; sICH, symptomatic intracranial hemorrhage.

This is a retrospective analysis of consecutive AIS patients with large vessel occlusion who underwent EVT from March 2016 to August 2023 in Figure 1. We included patients based on the following criteria: (1) patients who underwent thrombectomy employing second‐generation stent‐retriever devices or aspirators (Solitaire AB/FR, Covidien/ev3, Irvine, CA; Trevo Proview, Stryker, CA); (2) patients with occlusion of the large artery defined by digital subtraction angiography (DSA), including internal carotid artery (ICA), middle cerebral artery (MCA M1; M2), basilar artery (BA) and vertebral plus BA (VPBA); (3) mRS score before the index stroke ≤ 1; (4) EVT could be administered within 6 h after symptom onset or within 6–24 h after symptom onset with the presence of large ischemic mismatch/penumbra according to CT perfusion [20].

The exclusion criteria were as follows: (1) presence of intracranial hemorrhage identified on CT scan prior to EVT; (2) severe cardiac, hepatic, or renal insufficiency, or advanced diabetes mellitus with blood glucose levels exceeding 22 mmol/L; (3) coagulation disorders, such as platelet counts below 100 × 10^9^/L, or concurrent thrombotic conditions including but not limited to deep vein thrombosis, pulmonary embolism, and disseminated intravascular coagulation; (4) inability to obtain regular coagulation function tests within a 24‐h window preceding the operation or to compile complete medical records; and (5) lack of follow‐up imaging examinations or mRS scores after 3 months post‐EVT. This rigorous selection process ensures a homogeneous study population that is representative of clinical practice while minimizing confounding factors that could influence the study outcomes.

### 2.2 Data Collection

Data for our study were extracted from the electronic medical records system, which is supported by the Stroke Center at the Department of the Second Affiliated Hospital, Zhejiang University. The patients included in our study underwent EVT, encompassing a range of procedures such as thrombectomy with stent retrievers, thrombus aspiration, intracranial angioplasty, stent implantation, or any combination thereof, as determined by the treating surgeon. The occlusion sites of the arteries were identified through computed tomography angiography (CTA), magnetic resonance angiography (MRA), and/or cerebral DSA reports, and included vessels such as the ICA, MCA, BA, and vertebral artery (VERT).

The collected data were categorized into several domains, including baseline demographic data, medical history, stroke characteristics, procedural variables, treatment variables, and hospitalization complications, totaling 49 variables. Baseline characteristics encompassed age, sex, NIHSS score, and modified Rankin scale (mRS) score at admission, as well as comorbidities such as diabetes, hypertension, atrial fibrillation, hyperlipidemia, and a history of stroke, along with the use of antiplatelet and anticoagulation medications. Procedural time variables included metrics such as time from puncture to reperfusion (TPR), time from onset to groin puncture (OTP), time from onset to reperfusion (OTR), time from onset to admission (OTA), time from onset to imaging (OTI), and time from imaging to puncture (ITP). Additionally, we recorded the use of tissue plasminogen activator (t‐PA), the number of retrieval attempts exceeding three, and rescue therapies including balloon angioplasty and stenting. Reperfusion status was assessed using the modified thrombolysis in cerebral infarction (mTICI) scale by surgeons during the operation, with mTICI grades better than 2B indicating successful recanalization. Hospitalization complications such as hematoma transformation (HT), symptomatic intracranial hemorrhage (sICH), respiratory failure, liver dysfunction, and pulmonary infection were also documented.

Blood samples were collected prior to the emergency EVT. Venous blood samples of 4–6 mL were obtained within 24 h postadmission. In cases where blood tests were conducted multiple times within this timeframe, the initial value was utilized for analysis. Coagulation function indicators, including D‐dimer, INR, PT, PTA, APTT, TT, and FIB, were measured using the Sysmex CA‐7000 Automated Coagulation Analyzer (Sysmex, Kobe, Japan) [21].

### 2.3 Follow‐Up and Outcomes

The follow‐up protocol was consistent with previously published literature [22]. The primary outcome was good functional outcome at 90 days, defined as a mRS score of 0–3 [18]. The scores were collected by a stroke neurologist during routine follow‐up visits at 90 days (±14) after stroke for the majority of patients through telephone discussion or clinical follow‐up with patients or their families. As for complications, HT refers to the bleeding caused by the reperfusion of blood vessels in the ischemic area after acute cerebral infarction [23]. sICH was defined as any intracranial hemorrhage with an increase in the NIHSS score of ≥ 4 from baseline within 7 days after EVT, according to the ECASS (European–Australasian Acute Stroke Study) II criteria [23].

### 2.4 Statistical Analysis

Patients were dichotomized by favorable or unfavorable outcome groups based on mRS score at 3 months after EVT was 0–3 or 4–6. Details on the missing data are shown in Figure S1 without data missing more than 20%. For missing data sets, we use multiple interpolations to fill in the missing values. Characteristics are summarized as proportions for categorical variables and mean ± SD or median (25–75th percentile) for quantitative variables, as appropriate. Two‐sample *t*‐tests or Mann–Whitney *U* test was used to compare the dichotomous in continuous quantitative variables, while *χ* ^2^ tests or Fisher's exact test was used for categorical variables. Through ANOVA and univariate logistic regression, potential risk factors were filtrated to be associated with outcome of AIS patients (*p* < 0.05), and then these potential predictors were corrected by confounding factors which were filtrated from clinical characters through univariate logistic regression (*p* < 0.05). Results were displayed as adjusted odds ratios (OR) combined with a 95% CI. The ROC was used to determine the cut‐off value of predictors and their accuracy for the prediction of short‐medium prognosis. Subgroup analysis was conducted to further analyze the different prognostic efficacy of coagulation functions in certain subgroups. *p* values of < 0.05 were considered statistically significant. All reported *p* values were two sided. All statistical analyses were performed using IBM SPSS Statistics for Windows version 26 and R language 4.0.5.

## 3 Results

### 3.1 Baseline Characteristics of the Cohort

The patient characteristics are detailed in Table 1. Our final cohort comprised 607 patients with a mean age of 69.10 ± 13.22 years. Of these, 259 (42.67%) were female, and 359 (59.14%) had received IVT prior to EVT. The median NIHSS score at admission was 15 (IQR, 11–20), and the median mRS score was 5 (IQR 3–5). On average, thrombectomy involved two pass attempts (IQR 1–3). At the conclusion of thrombectomy, 562 (92.59%) patients achieved successful recanalization, defined by a mTICI score of 2B or 3.

**TABLE 1:** Baseline characteristics of patients with favorable or unfavorable outcomes after 3 months for endovascular treatment of acute large artery occlusive ischemic stroke.

Variables	All patients (*n* = 607)	Good functional outcome (*n* = 272)	Poor functional outcome (*n* = 335)	*p*
*Demographic characteristics*				
**Age, mean ± SD**	69.10 ± 13.22	64.62 ± 13.70	72.74 ± 11.62	< 0.001**
BMI, mean ± SD	23.37 ± 3.44	23.68 ± 3.33	23.13 ± 3.51	0.054
**Female, *n* (%)**	259 (42.67)	104 (38.24)	155 (46.27)	0.047*
**Age > 80, *n* (%)**	136 (22.41)	29 (10.66)	107 (31.94)	< 0.001**
**SBP, M (Q**_**1**_, **Q**_**3**_ **)**	145.00 (128.00, 162.00)	143.00 (124.75, 159.00)	148.00 (129.00, 165.00)	**0.022**
CBP, M (Q_1_, Q_3_)	81.00 (72.00, 91.00)	80.50 (72.00, 90.00)	82.00 (71.00, 92.00)	0.73
*Stroke characteristics*				
**NIHSS at admission, M (Q**_**1**_, **Q**_**3**_ **)**	15.00 (11.00, 20.00)	13.00 (9.00, 17.00)	17.00 (12.00, 24.00)	< 0.001**
**NHISS > 14, *n* (%)**	401 (66.06)	149 (54.78)	252 (75.22)	< 0.001**
**mRS at admission, M (Q**_**1**_, **Q**_**3**_ **)**	5.00 (3.00, 5.00)	3.00 (2.00, 4.00)	5.00 (5.00, 5.00)	< 0.001**
**TOAST classification, *n* (%)**				
LAA	276 (46.85)	140 (52.63)	136 (42.11)	0.218
CE	286 (48.56)	110 (41.35)	176 (54.49)	
Others or undetermined	18 (2.97)	6 (2.21)	12 (3.58)	
**Site of occlusion, *n* (%)**				
ICA	108 (17.79)	43 (15.81)	65 (19.40)	< 0.001*
MCA	319 (52.55)	168 (61.76)	151 (45.07)	
ICA‐MCA	64 (10.54)	23 (8.46)	41 (12.24)	
VERT/BA	79 (13.01)	19 (6.99)	60 (17.91)	
Others	37 (6.10)	19 (6.99)	18 (5.37)	
Multistage thrombus, *n* (%)	106 (17.46)	43 (15.81)	63 (18.81)	0.333
*Procedural variables*				
OTA, M (Q_1_, Q_3_)	266.00 (170.00, 374.00)	256.00 (170.00, 373.00)	270.50 (170.50, 375.75)	0.967
OTI, M (Q_1_, Q_3_)	218.00 (115.75, 332.25)	222.00 (124.00, 321.00)	217.00 (111.50, 338.00)	0.683
ITP, M (Q_1_, Q_3_)	104.00 (76.00, 138.00)	99.00 (76.00, 130.50)	107.27 (76.00, 141.00)	0.142
**PTR, M (Q**_**1**_, **Q**_**3**_ **)**	70.00 (45.00, 105.72)	63.00 (43.60, 96.00)	75.00 (50.00, 113.50)	0.005*
OTR, M (Q_1_, Q_3_)	400.00 (300.00, 521.00)	391.00 (296.50, 509.00)	413.50 (305.00, 529.50)	0.087
OTP, M (Q_1_, Q_3_)	325.00 (230.00, 431.50)	320.00 (230.44, 419.01)	330.50 (230.00, 440.00)	0.431
**Numbers of pass attempts, M (Q**_**1**_, **Q**_**3**_ **)**	2.00 (1.00, 3.00)	2.00 (1.00, 3.00)	2.00 (1.00, 3.00)	0.007*
**Numbers of pass attempts > 3, *n* (%)**	98 (16.14)	34 (12.50)	64 (19.10)	0.028*
**Successful recanalization (mTICI ≥ 2B/3), *n* (%)**	562 (92.59)	259 (95.22)	303 (90.45)	0.026*
*Treatment variable*				
Rescue therapy^a^	105 (17.30)	50 (18.38)	55 (16.42)	0.525
Tirofiban treatment, *n* (%)	105 (17.30)	56 (20.59)	49 (14.63)	0.053
Distal escape, *n* (%)	42 (6.92)	20 (7.35)	22 (6.57)	0.704
IVT, *n* (%)	359 (59.14)	165 (60.66)	194 (57.91)	0.493
**General anesthesia, *n* (%)**	311 (51.24)	126 (46.32)	185 (55.22)	0.029*
*Medical history*				
CIA, *n* (%)	134 (22.08)	53 (19.49)	81 (24.18)	0.166
Hyperlipemia, *n* (%)	22 (3.62)	10 (3.68)	12 (3.58)	0.951
**Hypertension, *n* (%)**	391 (64.42)	152 (55.88)	239 (71.34)	< 0.001**
**CHD, *n* (%)**	75 (12.36)	24 (8.82)	51 (15.22)	0.017*
**Diabetes, *n* (%)**	114 (18.78)	32 (11.76)	82 (24.48)	< 0.001**
**Atrial fibrillation, *n* (%)**	231 (38.06)	86 (31.62)	145 (43.28)	0.003*
Pre‐antiplatelet, *n* (%)	72 (11.86)	36 (13.24)	36 (10.75)	0.346
Pre‐anticoagulation, *n* (%)	84 (13.84)	35 (12.87)	49 (14.63)	0.532
Operation history, *n* (%)	205 (33.77)	83 (30.51)	122 (36.42)	0.126
*Hospitalization complications*				
**HT, *n* (%)**	205 (33.77)	51 (18.75)	154 (45.97)	< 0.001**
**sICH, *n* (%)**	133 (21.91)	4 (1.47)	129 (38.51)	< 0.001**
**Pulmonary infection, *n* (%)**	282 (46.46)	103 (37.87)	179 (53.43)	< 0.001**
Liver dysfunction, *n* (%)	74 (12.19)	32 (11.76)	42 (12.54)	0.772
**Respiratory failure, *n* (%)**	53 (8.73)	7 (2.57)	46 (13.73)	< 0.001**
*Coagulation function*				
**D‐dimer**	970.00 (505.00, 2205.00)	665.00 (380.00, 1472.50)	1290.00 (630.00, 2690.00)	**< 0.001**
**INR, M (Q**_**1**_, **Q**_**3**_ **)**	1.04 (0.98, 1.11)	1.02 (0.96, 1.08)	1.05 (0.98, 1.13)	**0.002**
APTT, M (Q_1_, Q_3_)	33.90 (31.25, 37.15)	33.90 (31.20, 36.82)	33.80 (31.30, 37.45)	0.731
TT, M (Q_1_, Q_3_)	17.20 (16.30, 18.20)	17.10 (16.20, 18.20)	17.20 (16.40, 18.20)	0.527
**PT, M (Q**_**1**_, **Q**_**3**_ **)**	13.40 (12.90, 14.20)	13.30 (12.80, 13.90)	13.50 (13.00, 14.30)	0.002*
**PTA, M (Q**_**1**_, **Q**_**3**_ **)**	94.00 (84.00, 104.00)	96.00 (87.00, 106.00)	93.00 (82.00, 103.00)	0.004*
**FIB, M (Q**_**1**_, **Q**_**3**_ **)**	3.07 (2.63, 3.67)	2.96 (2.53, 3.49)	3.18 (2.67, 3.82)	0.002*

*Note:* Qualitative variable are *n* (%), mean ± SD, and median (interquartile range). Bold values denote statistical significance at the *p* < 0.05 level.

Abbreviations: APTT, activated partial thromboplastin time; CE, cardioembolism; CHD, coronary heart disease; FIB, fibrinogen; HT, hemorrhagic transformation; ICA, internal carotid artery; INR, international normalized ratio; ITP, time from image to puncture; IVT, intravenous thrombolysis; LAA, large artery atherosclerosis; MCA, middle cerebral artery; MRS, modified Rankin scale; mTICI, modified thrombolysis in cerebral Infarction; NIHSS, National Institutes of Health Stroke Scale; OTA, time from onset to admission; OTI, time from onset to image; OTP, time from onset to groin puncture; OTR, time from onset to reperfusion; PT, prothrombin time; PTA, prothrombin time activity; sICH, symptomatic intracranial hemorrhage; TPR, time from puncture to reperfusion; TT, thrombin time.

^a^ Rescue therapy included balloon angioplasty and permanent stenting.

*p < 0.05; **p < 0.001.

Within the cohort, 335 (55.19%) AIS‐LVO patients exhibited an unfavorable outcome, characterized by an inability to walk independently 3 months later. Those with an unfavorable prognosis were older on average (72.74 vs. 64.62 years), more frequently female (46.27% vs. 38.24%), had higher systolic blood pressure (SBP) at emergency presentation (median 148 vs. 143 mmHg), and experienced longer times from puncture to recanalization (median 75 vs. 63 min). They also presented with higher NIHSS scores (median 17 vs. 13) and mRS scores (median 5 vs. 3). The prevalence of comorbidities was higher among patients with poor outcomes, including hypertension (13.73% vs. 2.57%), atrial fibrillation (43.28% vs. 31.62%), coronary heart disease (CHD) (15.22% vs. 8.82%), and diabetes (24.48% vs. 11.76%). Hospitalization complications were also more prevalent in this group, with higher rates of HT (45.97% vs. 18.75%), sICH (38.51% vs. 1.47%), pulmonary infection (53.43% vs. 37.87%), and respiratory failure (13.73% vs. 2.57%). In terms of coagulation function, patients with unfavorable prognosis had higher level of D‐dimer (median 1290 vs. 665 μg/L), INR (median 1.05 vs. 1.02), PT (median 13.5 vs. 13.3 s), and PFI (median 3.18 vs. 2.96 g/L) but lower PTA (median 93% vs. 96%) compared to those with favorable prognosis after EVT (Table 1). Other baseline characteristics and coagulation function indicators, like stroke etiology or APTT, did not have significant differences between two different groups (*p* > 0.05).

### 3.2 Adjusted Odds Ratio of D‐Dimer, INR, PT, and PFI With Poor Prognosis at 3 Months After EVT

After adjusting for variables found to be significant in univariate logistic analysis, we ensured that the tolerance of the variables included in the multivariate logistic analysis was greater than 0.1, and the variance inflation factor (VIF) was significantly less than 10 for all predictors. This indicated the absence of multicollinearity within our models. Elevated levels of D‐dimer (aOR = 1.01; 95% 1.01–1.01) and decreased PTA (aOR = 0.99; 95% CI 0.98–0.99) were significantly associated with a poor outcome in patients with AIS‐LVO following EVT (Table 2). Additionally, an increase in the INR showed a trend toward an increased risk of a poor prognosis, although this did not reach statistical significance (aOR = 2.14; 95% CI 0.93–4.96, *p* = 0.074). In the adjusted clinical variables, age above 80 years (aOR = 3.89; 95% CI 2.25–6.73), NIHSS score above 14 (aOR = 2.28; 95% CI 1.50–3.45), diabetes (aOR = 2.45; 95% CI 1.43–4.20), BA occlusion (aOR = 3.08; 95% CI 1.43–6.61), and atrial fibrillation (aOR = 1.53; 95% CI 1.01–2.34) were independently associated with a poor prognosis (Table S1).

**TABLE 2:** Unadjusted and adjusted ORs of the association of coagulation function indicators with poor outcome at 3 months after endovascular treatment.

Variables	Univariate		Multivariate		
	OR (95% CI)	*p*	*aβ*	aOR (95% CI)	*ap*
**D‐dimer**	1.01 (1.01–1.01)	0.006	0.01	1.01 (1.01–1.01)	0.011*
INR	1.98 (1.01–3.86)	0.045	0.76	2.14 (0.93–4.96)	0.074
PT	1.00 (0.98–1.02)	0.803	**—**		
**PTA**	0.99 (0.98–0.99)	0.009	−0.01	0.99 (0.98–0.99)	0.017*
FIB	1.29 (1.09–1.53)	0.003	0.15	1.16 (0.96–1.41)	0.131

*Note:* Adjust procedural variables incorporate male, age higher than 80 years, successful recanalization (mTICI = 2B or 3), general anesthesia, NHISS higher than 14, hypertension, CHD, diabetes, site of occlusion, atrial fibrillation, pass attempts over three times, body fat index, PTR, and baseline SBP. Bold values denote statistical significance at the *p* < 0.05 level.

Abbreviations: CI, confidence interval; OR, odds ratio.

*p < 0.05; **p < 0.001.

### 3.3 Diagnostic Efficiency of D‐Dimer and PTA With Operating Characteristic Curve

**FIGURE 2:**
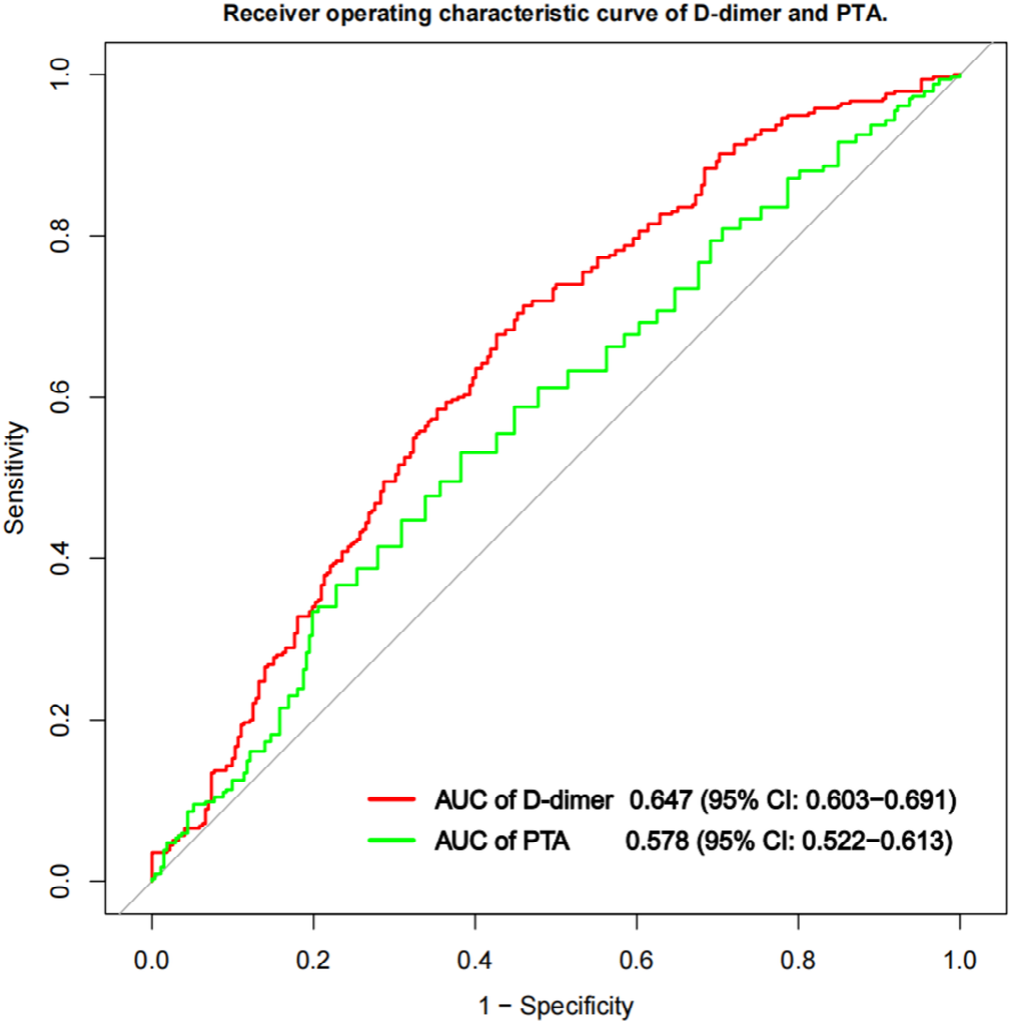
Receiver operating characteristic (ROC) curve was used to evaluate the predictive ability of plasma D‐dimer and PTA levels for 3 months of unfavorable prognosis of AIS patients after EVT (area under the curve = 0.647 and 0.578, respectively).

Based on the receiver operating characteristic curve (ROC) in Figure 2, the optimal cut‐off value of D‐dimer as a predictor for poor outcome was 715 mg/L, which showed a sensitivity of 71.3% and a specificity of 54.1% with an AUC of 0.647 (95% CI 0.603–0.692). In the same way, the optimal cut‐off value of PTA was 85.5% with a sensitivity of 79.3%, a specificity of 33.4%, and an AUC of 0.578 (95% CI, 0.522–0.613). Through cut‐off value, we further divided patients into the low D‐dimer group (D‐dimer < 715 mg/L) and the elevated D‐dimer group (D‐dimer ≥ 715 mg/L). Similarly, we also divided patients into low PTA group (PTA < 85.5%) and the elevated PTA group (PTA ≥ 85.5%). The clinical characteristics of patients with normal and elevated groups are shown in Table S2. What is more, after adjustment for significant variables in univariate analysis, male (acOR = 0.61; 95% CI 0.39–0.96), BA occlusion (acOR = 0.32; 95% CI 0.12–0.84), and baseline mRS score (acOR = 1.24; 95% CI 1.06–1.46) were significantly associated with baseline elevated D‐dimer levels (Table S3). For emergency PTA, CE stroke etiology (acOR = 0.43; 95% CI 0.25–0.77), operation history (acOR = 0.4; 95% CI 0.24–0.67), and history of taking anticoagulant drugs (acOR = 0.26; 95% CI 0.14–0.5) were significantly associated with baseline elevated PTA after correcting for confounding variables (Table S4).

We further explore the association between elevated D‐dimer and PTA group and poor prognosis of AIS in Table 3. After adjusting for other covariates, elevated D‐dimer was the independent predictor of poor prognosis, with 2.51 times higher risk of poor outcome in elevated D‐dimer (≥ 715 mg/L) than normal D‐dimer group (aOR = 2.51, 95% CI 1.59–3.97). However, the elevated PTA (≥ 85.5%) was independently and inversely linked to poor prognosis, and the risk of poor prognosis was 0.39 times in the elevated PTA group than the normal group (aOR = 0.39, 95% CI 0.18–0.87). Elevated D‐dimer and low PTA possessed the synergistic effect, presenting the strongest protective effect on unfavorable prognosis of patients with AIS‐LVO after EVT, compared with elevated D‐dimer or PTA group (aOR = 0.22, 95% CI 0.13–0.38). The relationship between D‐dimer and PTA and long‐term prognosis at 12 months after EVT was similar to results of prognosis at 3 months. Besides, D‐dimer and PTA also present synergistic effects, with low D‐dimer but elevated PTA group exhibiting the strongest protective effects for long‐term prognosis in Table S5 (aOR = 0.19, 95% CI 0.09–0.37).

**TABLE 3:** Unadjusted and adjusted ORs of the association of D‐Dimer and PTA with poor outcome at 3 months after endovascular treatment.

Grouping variable	Univariate		Multivariate		
	OR (95% CI)	*p*	*aβ*	aOR (95% CI)	*ap*
**Elevated D‐dimer group**	2.93 (2.09–4.10)	< 0.001	0.92	2.51 (1.59–3.97)	< 0.001**
**Elevated PTA group**	0.52 (0.36–0.75)	< 0.001	−0.93	0.39 (0.18–0.87)	0.02*
*Ddimer and PTA classification*					
Elevated D‐dimer but low PTA	1.00 (Reference)			1.00 (Reference)	
**Elevated D‐dimer and PTA**	0.62 (0.39–0.99)	0.047	−0.54	0.59 (0.35–0.98)	0.042*
**Low D‐dimer and PTA**	0.34 (0.16–0.73)	0.005	−0.89	0.41 (0.18–0.96)	0.039*
**Low D‐dimer but elevated PTA**	0.24 (0.15–0.38)	< 0.001	−1.51	0.22 (0.13–0.38)	< 0.001**

*Note:* Adjust procedural variables incorporate male, age higher than 80 years, successful recanalization (mTICI = 2B or 3), general anesthesia, symptomatic cerebral hemorrhage, pulmonary infection, respiratory failure, NHISS higher than 14, hypertension, CHD, diabetes, site of occlusion, atrial fibrillation, pass attempts over three times, body fat index, PTR, and baseline SBP. Bold values denote statistical significance at the *p* < 0.05 level.

Abbreviations: CI, confidence interval; OR, odds ratio.

*p < 0.05; **p < 0.001.

### 3.4 Subgroup Analysis of Elevated D‐Dimer and PTA Group

**FIGURE 3:**
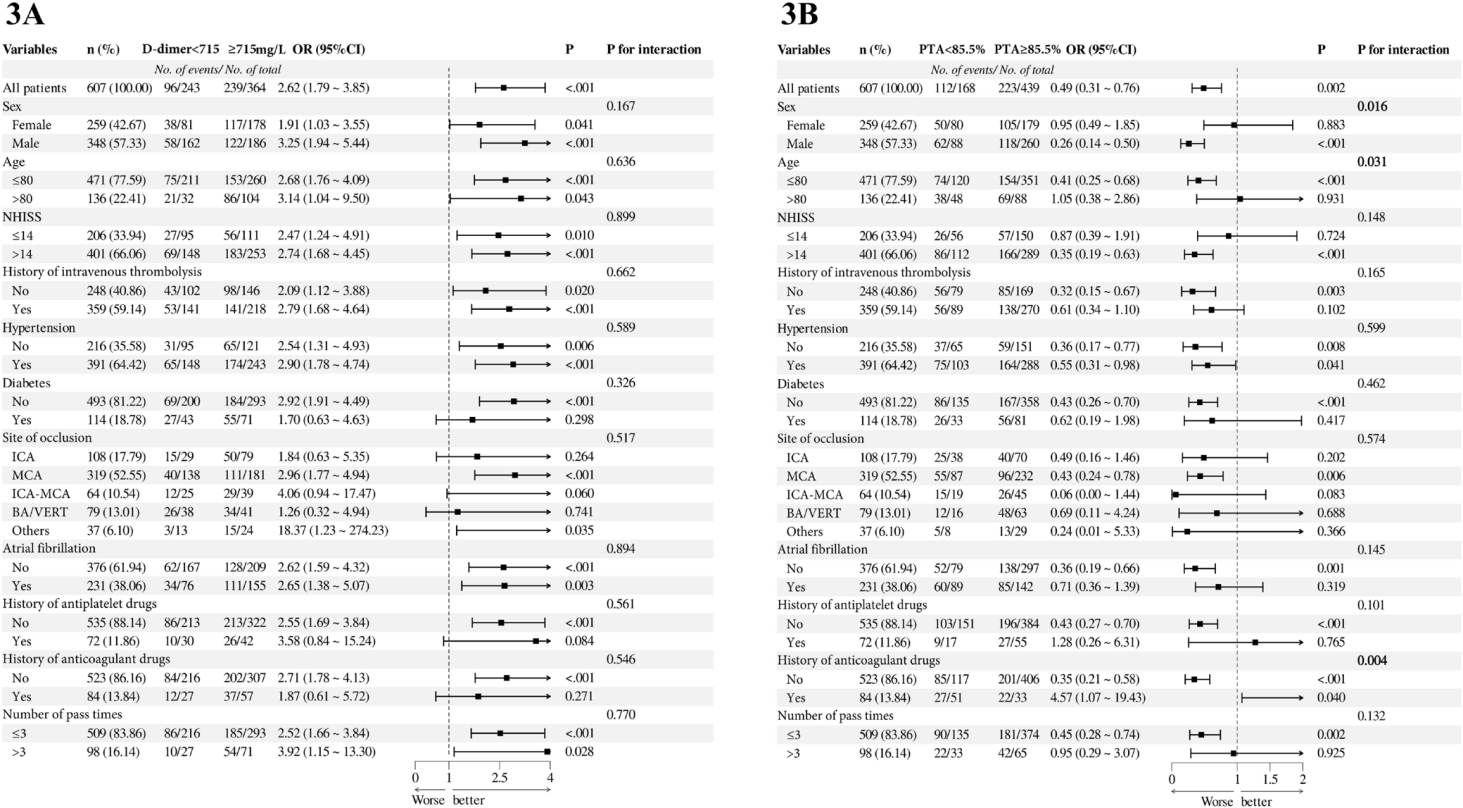
Subgroup analysis of elevated D‐dimer and PTA group for prognosis at 3 months after EVT. (A) Subgroup analysis of unfavorable prognosis in elevated D‐dimer group. (B) Subgroup analysis of unfavorable prognosis in elevated PTA group. Confounding factors included in correction include sex, age, NHISS score, history of intravenous thrombolysis, hypertension, diabetes, site of occlusion, atrial fibrillation, history of antiplatelet drugs, history of anticoagulant drugs, and number of pass times.

Subgroup variables were selected from positive clinical risk factors after multivariate correction (Figure 3). We found that the increased D‐dimer was an independent risk factor for poor prognosis at 3 months after EVT in patients with AIS‐LVO, compared with the D‐dimer less than 715 mg/L, regardless of the subgroup of vascular recanalization, NHISS score, preoperative thrombolysis, hypertension, atrial fibrillation, and number of attempt times, which further expand the generalizability and credibility of the conclusions. However, there was no interaction within these subgroups (*p* for interaction > 0.05). As a protective factor for prognosis, in a certain subgroup of males, age less than 80 years, NHISS score greater than 14, direct mechanical thrombectomy (MT), passes times less than 3, and without hypertension, diabetes, atrial fibrillation, or history of anticoagulant and antiplatelet drugs before onset, higher PTA (≥ 85.5%) was significantly associated with favorable prognosis after EVT. At the same time, significant interaction effects were shown in the three subgroups of gender, age, and history of anticoagulant drug use (*p* for interaction < 0.05), suggesting the importance of controlling PTA in these three subgroups, which may be beneficial to understanding the mechanisms by which PTA affects prognosis.

## 4 Discussion

In clinical guidelines, the key to the treatment of AIS is rapid restoration of blood supply to ischemic brain tissue as soon as possible and salvage ischemic penumbra [24]. Nowadays, with more updated retriever devices and various kinds of thrombectomy techniques having been stabilized, the successful recanalization proportions of EVT are between 83% and 92% [25]. In the latest clinical trials, results showed even higher recanalization rates with up to 95% of patients achieving successful recanalization [18]. However, the rate of favorable clinical outcomes still wanders around 50% even lower [26]. Therefore, it is urgent and important to explore which factors cause and influence the mismatch between technical success rates and clinical outcomes, which will be beneficial in guiding clinical treatment decisions and making personalized treatment plans according to different conditions of patients and their families. As a routine clinical preoperative examination, we systemically investigated the associations between coagulation function profile (PT, INR, PTA, TT, APTT, and FIB) and unfavorable prognosis of patients with AIS at 3 months after EVT. After adjusting for other covariates, D‐dimer was the independent predictor of poor prognosis, with 2.51 times higher the risk of poor outcome in elevated D‐dimer group than low D‐dimer group (aOR = 2.51, 95% CI 1.59–3.97). However, high level of PTA was independently and inversely linked to poor prognosis, and the risk of poor prognosis was 0.39 times higher in the elevated PTA group than in the normal group (aOR = 0.39, 95% CI 0.18–0.87). In addition, elevated D‐dimer and low PTA had synergistic effect, presenting higher association with poor outcome of patients with AIS‐LVO (aOR = 4.55, 95% CI 2.63–7.69). The increase in INR had a tendency to increase the risk of poor prognosis (*p* = 0.074), and no correction between PT, APTT, and FIB and poor outcome was found.

Subgroup analysis showed that three subgroups, incorporating failed recanalization, complicated with diabetes and not the MCA occlusive affected the predictive efficacy of both D‐dimer and PTA for clinical outcome, which deserve more attention in clinical practice. Although D‐dimer was significantly associated with poor prognosis in multiple subgroups, with good universality and confidence, it may suggest that real‐time monitoring of D‐dimer level can effectively predict the risk of adverse prognosis in patients in advance. However, there was no interaction effect within subgroups and certain specificity was lacking. Preoperative D‐dimer demonstrated a significant association with short‐ and long‐term prognosis after EVT across multiple subgroups, indicating strong universality and confidence in its predictive value. This suggests that real‐time D‐dimer monitoring could serve as an effective tool for preemptively identifying patients at risk of adverse outcomes. However, the absence of interaction effects within subgroups and the lack of specificity highlight areas for further research. For PTA, in the subgroup of males, age younger than 80 years, and without history of anticoagulant drug use, the high PTA group (> 85.5%) was significantly associated with favorable prognosis of patients with AIS‐LVO than the low PTA group, with significant interaction effects. This underscores the value of PTA control in this specific demographic and its potential role in prognosis.

As a soluble fibrin degradation product that results from ordered breakdown of thrombi by the fibrinolytic system, D‐dimer exhibits a close connection with AIS. Numerous studies have shown that D‐dimer serves as a valuable marker of activation of coagulation and fibrinolysis [27]. The increase in D‐dimer concentration is a sensitive marker of fibrinolysis secondary to acute thrombosis, which is closely related to the occurrence, development, and prognosis of stroke [28]. In ischemic stroke, the baseline elevated D‐dimer levels may reflect ongoing microthrombosis in the target vessel occlusion territory, thus causing extensive activation of the coagulation system, subsequent hyperfibrinolysis, and coagulopathic disturbances [18]. Han et al. [29] reported that AIS patients are at increased risk of developing DVT following EVT, particularly if they have undergone prolonged thrombectomy procedures and exhibit high plasma levels of D‐dimer (aOR = 1.35, 95% CI 1.15–1.59). Wang et al. [30] demonstrated that D‐dimer levels before reperfusion were independent predictors for 3‐month unfavorable outcomes in AIS patients undergoing reperfusion therapy (no matter IVT and/or endovascular). In atrial fibrillation patients, D‐dimer‐to‐FIB ratio independently predicts early neurological deterioration (END) in ischemic stroke with adjusted odds ratio (aOR = 2.14, 95% CI 1.24–3.6) [31]. More importantly, a latest study demonstrates that elevated admission of D‐dimer level was an independent predictor of sICH in patients with AIS after thrombectomy (aOR = 2.45, 95% CI 1.75–3.43). Based on the ROC, the Ddimer, as a predictor for predicting sICH, presented with a specificity of 86.2% and an area under the curve of 0.774 [18]. Besides, it has been demonstrated that HT, especially sICH, plays a counteract role in functional outcomes of AIS following EVT [26]. So, D‐dimer levels may indirectly influence patients' prognosis of 3 months by influencing the occurrence of procedure complications.

PTA is an important indicator reflecting the synthesis function of coagulation factors in the liver, which is usually done by a functional measurement of prothrombin in plasma, reflecting the actual functional level of prothrombin in blood clotting [17]. When liver hepatocytes are seriously damaged, the reduction in coagulation factor synthesis PTA will decrease. Therefore, the level of PTA has been reported that can reflect the degree of hepatocyte damage, which is of great value for the condition evaluation and prognosis of patients with liver failure [32]. No previous studies have reported the prognostic effect of PTA in stroke patients. Our study found that the level of PTA was a protective factor for ischemic stroke prognosis. The risk of poor prognosis in AIS‐LVO patients after EVT with elevated PTA (> 85.5%) was 0.39 times compared with that in patients with low PTA group (aOR = 0.39, 95% CI 0.18–0.87), showing liver function may indirectly affect the prognosis of stroke patients. As the indicator of the liver's ability to produce coagulation factors, effective hepatoprotective treatment and enhancement of the liver's capacity to produce coagulation factors may become a new method to assist in the functional recovery of stroke patients in clinical practices.

PT is a common index to assess the status of exogenous coagulation function, which measures the time of activity of clotting factors in the blood, specifically the time of activation of clotting factor I (prothrombin) into thrombin in vitro [21]. INR is closely related to PT, which is a standardized method of comparing a patient's PT results to an internationally standardized sample to eliminate variability between different laboratories, different reagents, and different test equipment [17]. In the present study, we found no association between PT/INR and poor prognosis of AIS patients after EVT, which was consistent with previous studies [33, 34]. As calculated by PT, INR directly reflects the status of blood coagulation and can be elevated by anticoagulation treatment [17]. In our cohort, proportion of patients with atrial fibrillation was 38.06%, which may affect hemodynamics and coagulation function to affect INR rate. Therefore, the extrinsic pathway of coagulation may not have a vital role in affecting prognosis, but therapeutic interventions that may increase level of PT and INR in AIS patients should be selected carefully. During blood clotting, FIB is activated together with prothrombin and converted into fibrin, forming a web of fibers that eventually leads to the formation of blood clots. Thrombin can cleave FIB into fibrin to form blood clots and exert impacts on fibrinolysis in different directions [35]. Furthermore, the structure of a fibrin clot was found related to functional outcomes in ischemic stroke patients [36]. In our research, we found that there is no association between FIB and poor prognosis. In our study, we only investigated the emergency level of FIB of patients with AIS‐LVO and found no association. However, as a dynamic, the FIB level will fluctuate in different time periods with the progression of ischemic region and blood reperfusion. Hence, the dynamic change in FIB and its role in the prognosis of AIS deserve further study.

In addition to D‐dimer and PTA, we also found some other factors, such as higher age, sex, use of tirofiban, and mRS at admission, are independently associated with prognosis of AIS. Previous studies have shown that the prognosis of elderly patients after MT is relatively poor. The reason is that most elderly patients take anticoagulants and antiplatelet drugs for a long time due to other diseases, which may lead to secondary cerebral hemorrhage [7]. In addition, due to atherosclerosis and intravascular plaque formation in elderly patients, the operation of MT is more difficult. In addition, the probability of complications is higher than that of younger patients, and the tolerance to surgery is worse than that of younger patients [37]. Studies have reported variable results for stroke case fatality by sex, with many providing little evidence of a difference, some showing higher case fatality, and others reporting lower case fatality for women. Overall, baseline differences in age, stroke characteristics, and cerebrovascular risk factors account for much of the observed sex differences in case fatality. Age‐adjusted mortality rates are commonly reported, but because sex differences are strongly modified by age, they can mask the complex relation of sex differences across age groups and can hide the burden of stroke mortality for elderly women [38].

There are still some limitations in our study: Firstly, it is based on single‐center data, which may not capture the full clinical profile and endovascular treatment trends for AIS‐LVO patients. Secondly, as a retrospective study, it is susceptible to selection bias, and therefore, the results should be interpreted cautiously. Thirdly, the time span of our cohort is long from 2016 to 2023, which may cause deviation due to the progress of materials and the maturity of surgical techniques to improve the treatment effect and prognosis. Finally, we did not track and analyze serial D‐dimer levels postthrombectomy, a parameter that could be key to understanding patient outcomes. Further studies are warranted to determine whether dynamic D‐dimer and PTA changes are also associated with the moderate and long‐term prognosis in AIS‐LVO. Exploring the pathophysiological mechanism of association between D‐dimer and PTA levels may be beneficial to explain the cause of poor functional prognosis of patients with AIS after thrombectomy.

## 5 Conclusions

In conclusion, our study systemically explored preoperative coagulation function and revealed that D‐dimer and PTA levels were independent predictors of 3‐month prognosis after thrombectomy in patients with AIS‐LVO. Besides, INR showed a tendency to increase the risk of poor prognosis but no significance was found. Elevated D‐dimer group (> 715 μg/L) was a risk factor for poor prognosis but the elevated PTA (≥ 85.5%) was a protective factor. In addition, elevated D‐dimer and low PTA had synergistic effect, presenting higher adjusted odds ratio with prognosis after 3 months. Through subgroupanalysis, we found that patients with failed recanalization, complicated with diabetes and not the MCA occlusive, these three factors that affect the predictive efficacy of both D‐dimer and PTA for clinical outcome, which deserve more attention in clinical practice. Given that quantitative measurements of D‐dimer and PTA levels are easily obtainable in clinical practice, their use as a blood biomarker predicting prognosis after EVT may add early prognostic information and facilitate perioperative management.

## Author Contributions

Conception and design: S.J. and P.G. Acquisition of data: C.Q., J.Y., and X.L. Analysis and interpretation of data: L.X. Drafting of the article: S.J. and P.G. Critically revising the article: F.B. and Z.T. Reviewed submitted version of manuscript: all authors. Approved the final version of the manuscript on behalf of all authors: J.L. and J.X. Administrative/technical/material support: X.C. and F.B. Study supervision: J.L.

## Ethics Statement

This large retrospective study was approved by Human Research Ethics Committee, the Second Affiliated Hospital of Zhejiang University School of Medicine. Without causing any potential harm to the patient, the patient's informed consent is exempt.

## Consent

The authors have nothing to report.

## Conflicts of Interest

The authors declare no conflicts of interest.

## Supporting information


Data S1.

## Data Availability

The data that support the findings of this study are available from the corresponding author upon reasonable request.
