# Supplementary material for: Emergency Admission Plasma D‐Dimer and Prothrombin Activity: Novel Predictors for Clinical Outcomes After Thrombectomy in Acute Ischemic Stroke With Large Artery Occlusion
Source: CNS Neurosci Ther. 2025 Feb 13;31(2):e70267. doi: 10.1111/cns.70267 (PMC11822457; doi:10.1111/cns.70267)
Supplement: Supplementary file 1 — Data S1. [file CNS-31-e70267-s001.docx]

**Online supplemental Figure 1.**


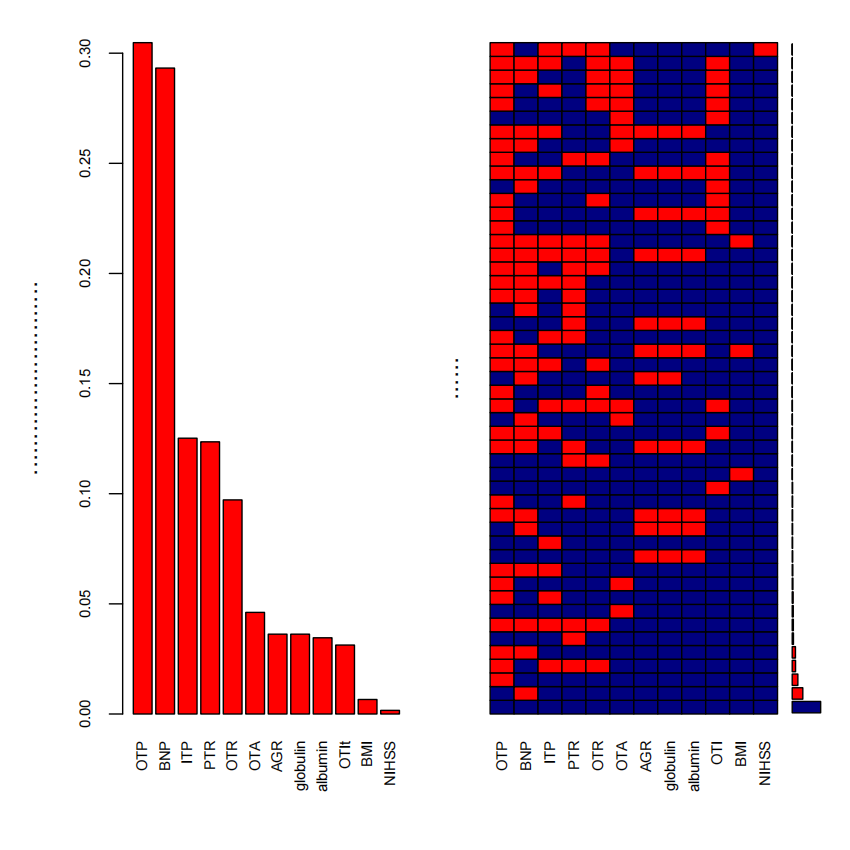


**Online supplemental Figure 2.**


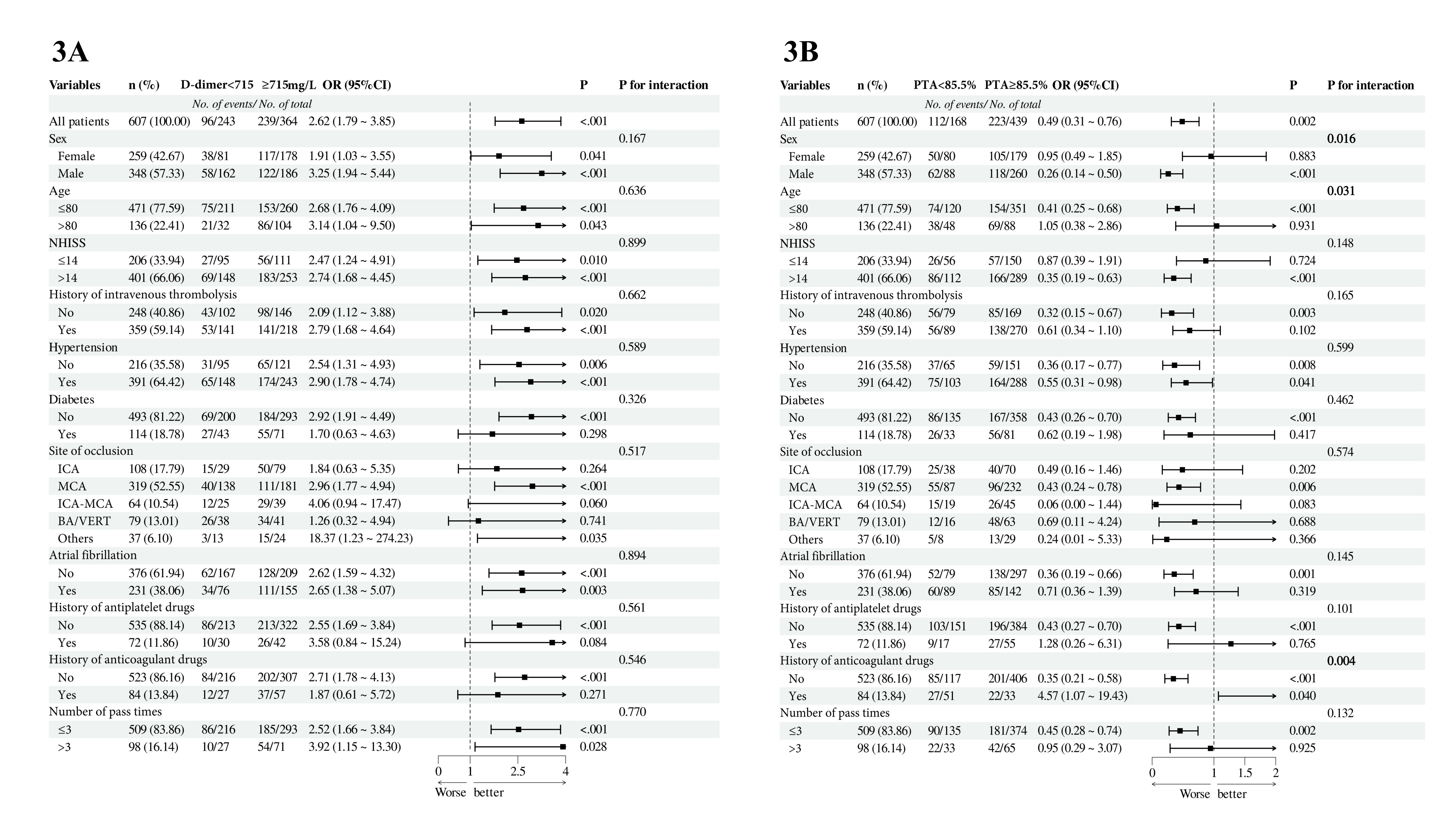


**Online supplemental Table 1.**

**Online supplemental Table 2.**


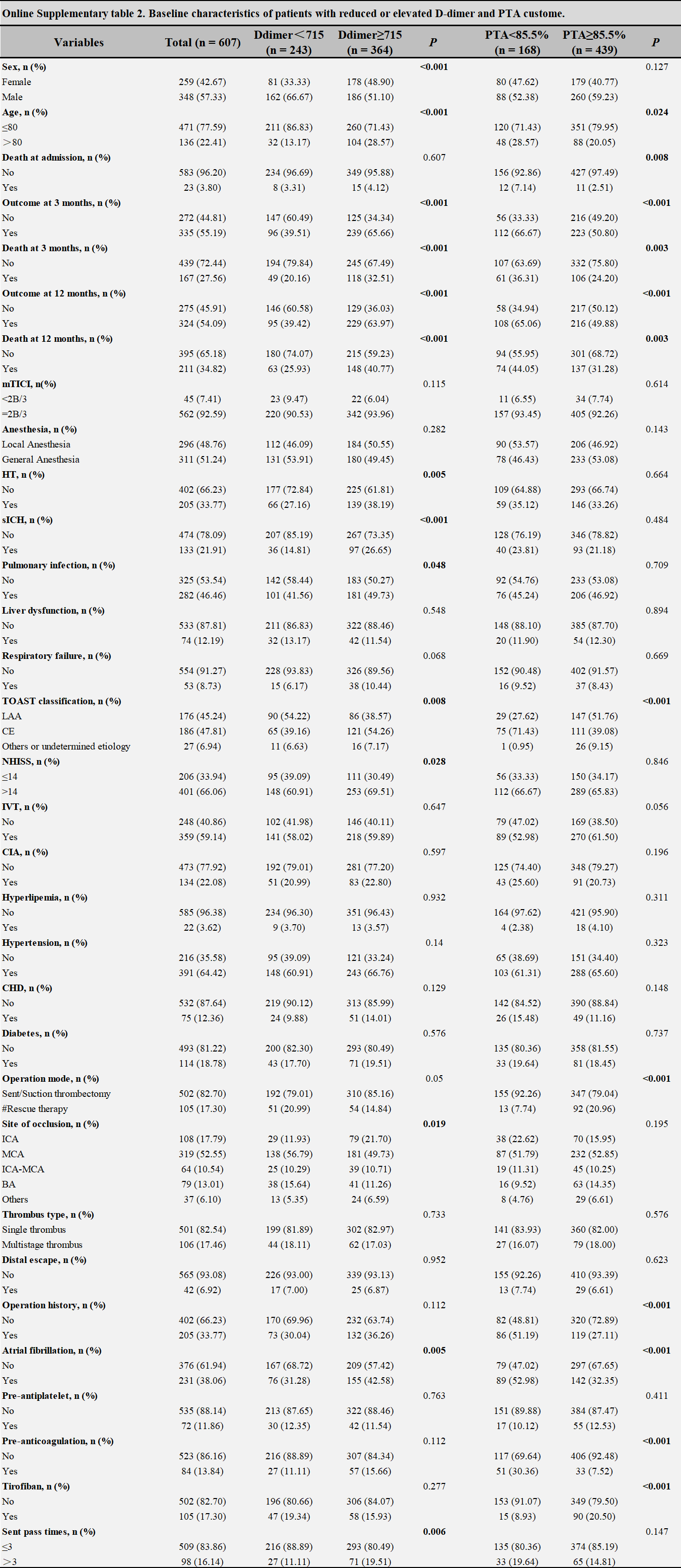


**Online supplemental Table 3.**

**Online supplemental Table 4.**

**Online supplemental Table 5.**
